# Pluronics-Formulated Farnesol Promotes Efficient Killing and Demonstrates Novel Interactions with *Streptococcus mutans* Biofilms

**DOI:** 10.1371/journal.pone.0133886

**Published:** 2015-07-29

**Authors:** Austin B. Mogen, Fu Chen, Sang-Joon Ahn, Robert A. Burne, Dong Wang, Kelly C. Rice

**Affiliations:** 1 Department of Microbiology and Cell Science, Institute of Food and Agricultural Sciences, University of Florida, Gainesville, Florida, 32611, United States of America; 2 Department of Pharmaceutical Sciences, College of Pharmacy, University of Nebraska Medical Center, Omaha, Nebraska, 68198, United States of America; 3 Department of Oral Biology, College of Dentistry, University of Florida, Gainesville, Florida, 32611, United States of America; LSU Health Sciences Center School of Dentistry, UNITED STATES

## Abstract

*Streptococcus mutans* is the primary causative agent of dental caries, one of the most prevalent diseases in the United States. Previously published studies have shown that Pluronic-based tooth-binding micelles carrying hydrophobic antimicrobials are extremely effective at inhibiting *S*. *mutans *biofilm growth on hydroxyapatite (HA). Interestingly, these studies also demonstrated that non-binding micelles (NBM) carrying antimicrobial also had an inhibitory effect, leading to the hypothesis that the Pluronic micelles themselves may interact with the biofilm. To explore this potential interaction, three different *S*. *mutans* strains were each grown as biofilm in tissue culture plates, either untreated or supplemented with NBM alone (P85), NBM containing farnesol (P85F), or farnesol alone (F). In each tested *S*. *mutans* strain, biomass was significantly decreased (SNK test, p < 0.05) in the P85F and F biofilms relative to untreated biofilms. Furthermore, the P85F biofilms formed large towers containing dead cells that were not observed in the other treatment conditions. Tower formation appeared to be specific to formulated farnesol, as this phenomenon was not observed in *S*. *mutans* biofilms grown with NBM containing triclosan. Parallel CFU/ml determinations revealed that biofilm growth in the presence of P85F resulted in a 3-log reduction in viability, whereas F decreased viability by less than 1-log. Wild-type biofilms grown in the absence of sucrose or *gtfBC* mutant biofilms grown in the presence of sucrose did not form towers. However, increased cell killing with P85F was still observed, suggesting that cell killing is independent of tower formation. Finally, repeated treatment of pre-formed biofilms with P85F was able to elicit a 2-log reduction in viability, whereas parallel treatment with F alone only reduced viability by 0.5-log. Collectively, these results suggest that Pluronics-formulated farnesol induces alterations in biofilm architecture, presumably via interaction with the sucrose-dependent biofilm matrix, and may be a viable treatment option in the prevention and treatment of pathogenic plaque biofilms.

## Introduction

Dental caries is well established as a biofilm-mediated disease, which if left untreated, can lead to tooth decay and severe patient suffering (pain, tooth loss, infection) [1, 2]. *Streptococcus mutans* is highly associated with the development of dental caries in humans [3, 4]. Formation of dental caries occurs through the fermentation of carbohydrates by acidogenic microorganisms, leading to the dissolution of mineral in enamel and dentin [5]. *S*. *mutans* is particularly cariogenic due to its highly acidogenic properties, ability to ferment sucrose, and production of insoluble extracellular polysaccharides (EPS), which aid in both adhesion and biofilm formation [6, 7]. One such group of exopolysaccharides are the sucrose-derived glucan polymers, a main component of the biofilm matrix, and synthesized by one of three glucosyltransferase enzymes (GtfB, GtfC, GtfD) [8]. Production of these extracellular polymers promotes adherence to the tooth surface [9–11], resistance to detachment by mechanical forces [12–14], and plays a primary role in the formation of dental caries [14, 15].

Currently established prevention and treatment of dental caries relies heavily on tooth-brushing and the strengthening of tooth enamel by fluoride. Antibiotics used in treatment of dental caries have also been pursued, although with somewhat limited effect. Chlorhexidine (CHX) gels and mouthwashes have demonstrated the most effectiveness in limiting dental plaque, but examination of CHX treatment across various subject groups showed inconclusive results [16, 17]. Testing of common antibiotics (erythromycin, penicillin, methicillin, ampicillin, and others) on a panel of 82 *S*. *mutans* strains (human, animal, and lab isolates) displayed mixed results, with many strains being resistant [18]. Additionally, most of these antibiotics are much less effective against biofilm cells and therefore cannot be confidently utilized as a primary treatment of dental caries [19–22]. Concerns regarding the emergence of antibiotic resistance among resident microbiota and the inherent recalcitrance of biofilms to antibiotics have therefore led to the exploration of alternative antimicrobial strategies for caries treatment.

Pluronics are surfactant copolymers comprised of both polyethylene oxide (PEO) and polypropylene oxide (PPO) blocks, with the balance of specific hydrophilic and lipophilic components dictating their amphiphilicity and surface activity [23]. Naturally present as unimers, Pluronics self-assemble into micelles when mixed in solutions above their critical micelle concentration (CMC) [24]. Consisting of a hydrophobic core and hydrophilic outer shell, considerable amounts (20–30% wt/vol) of water insoluble compounds can be packaged within these micelles [25]. For the purpose of inhibiting oral biofilm, our previous studies demonstrated that Pluronics could be chemically-conjugated to biomineral-binding moieties such as alendronate [26, 27], diphosphoserine peptides [28], and pyrophosphate [28], each of which displayed high binding affinity to hydroxyapatite (HA). These modified Pluronics were also successfully used to create tooth-binding micelles (TBMs), which when formulated with hydrophobic antimicrobials (triclosan, farnesol), were extremely effective at both inhibiting *S*. *mutans* biofilm formation on HA, as well as reducing viability of pre-formed biofilms [26–28]. Additionally, these studies demonstrated that unmodified Pluronic micelles carrying hydrophobic antimicrobials also had a modest inhibitory effect, suggesting that these “non-binding micelles” (NBMs) may also interact with *S*. *mutans* cells and/or biofilm. Therefore, this current study was undertaken to better understand the morphological changes and degree of cell death in *S*. *mutans* biofilms when co-cultured with NBMs containing hydrophobic antimicrobial compounds.

## Materials and Methods

### Bacterial strains and culturing conditions

The prototype *S*. *mutans* lab strain UA159 [29], UA159 *gtfBC* mutant (SAB109; this study), *S*. *mutans* BM71 [30] and *S*. *mutans* P1 [31] were maintained at -80°C in 25%-40% (vol/vol) glycerol. For each experiment, strains were freshly-streaked from frozen stocks onto Todd-Hewitt-Yeast Extract (THYE; Todd-Hewitt broth + 0.3% Yeast Extract) agar (supplemented with 1000 μg/ml kanamycin when culturing the *gtfBC* mutant), and incubated at 37°C, 5% CO_2_ for 48 hours.

### Creation of a UA159 *gtfBC* mutant

The *gtfBC*-deficient strain of *S*. *mutans* UA159, SAB109, was created using a PCR ligation mutagenesis approach [32] to replace nearly all of the open reading frame (7 nucleotides downstream of the *gtfB* start codon through 33 nucleotides upstream of the *gtfC* stop codon) with a polar kanamycin marker (ΩKm). Briefly, the upstream and downstream regions flanking *gtfBC* were amplified with primer pairs gftB-A (5’-CTGGGCTTGTTGCTGGAATC-3’)/ gftB-*BamHI*-B (5’-GTTTATAAC*GGATCC*TCT TGTCCAT-3’) and gftC-*BamHI*-C (5-CGTTTTAGA*GGATCC*CGTAATGGAT/ gftC-D (5’-CTCCTAACAGCGCTGCCATA-3’), respectively. These PCR fragments were then digested with BamHI and ligated to the kanamycin resistance cassette, and the mixture was transformed in the presence of competence stimulating peptide into *S*. *mutans* cells as previously-described [32]. Transformants were selected on BHI agar with kanamycin. Double-crossover recombination into each gene was confirmed by PCR and sequencing to ensure that no mutations were introduced into flanking genes.

### Pluronic micelle preparation and antimicrobial formulation

Pluronic P85 (molecular weight = 4600) or P123 (molecular weight = 5750) were used to prepare all non-binding micelle (NBM) formulations, as indicated. P85 and P123 unimers spontaneously form into micelles when mixed in solution at concentrations exceeding their specific CMCs (6.5x10^-5^ M and 4.4x10^-6^ M, respectively) [25]. In this study, all Pluronic solutions were prepared by first mixing either P85 or P123 in deionized water to a concentration of 2% (wt/vol) (4.3x10^-3^ M and 3.5 x 10^−3^ M, respectively), and dissolved overnight at 4°C. To prepare NBMs containing farnesol, each P85 or P123 solution was then warmed in a 37°C water bath for 30 minutes before adding farnesol to a final concentration of 1% (vol/vol). These solutions were then allowed to incubate overnight at 37°C with shaking at 105 RPM to allow for complete packaging of farnesol into the P85 or P123 micelles. Each solution was then filter sterilized using a 0.2 μm filter. Solutions of 1% (wt/vol) farnesol alone (F) were prepared in 100% (vol/vol) ethanol.

To prepare triclosan NBMs (P123T), a small magnetic stir bar was placed in a 5 ml P123 solution (prepared as described above), followed by agitation at high-speed on a magnetic stir plate and stepwise addition of 100 mg (2% wt/vol) triclosan over a 1 hour period. The P123 triclosan mixture was then continuously stirred overnight at room temperature, followed by centrifugation at 12,000 rpm for 30 seconds to remove any undissolved triclosan. The supernatant (containing P123 triclosan) was removed and filter sterilized with a 0.2 micron filter prior to experimental use.

### Confocal laser scanning microscopy (CLSM) of *S*. *mutans* biofilms grown in the presence of Pluronic micelles

Confocal microscopy was used to assess the effect of Pluronic micelles on *S*. *mutans* biofilm structure. In brief, overnight THYE broth cultures of each *S*. *mutans* strain were diluted to an OD_600_ of 0.01 in biofilm media (BM) [33] containing either 0.25% (wt/vol) glucose and 0.25% (wt/vol) sucrose (“sucrose-dependent” biofilms) or 0.5% (wt/vol) glucose (“sucrose-independent” biofilms). A 5 μl volume of each treatment stock solution (P85, P85F, P123, P123F, P123T or F alone) was then added to 1 ml of diluted culture, which was then vortexed for fifteen seconds and 200 μl transferred in duplicate to wells of a 96-well optically-clear tissue culture plate (Costar 3720). The final concentration of farnesol or triclosan in each treatment was 50 μg/ml or 36 μg/ml, respectively (each approximately 3.5× MIC). After 48 hours of static incubation at 37°C, 5% CO_2_, the media was removed from each well, and 200 μl BacLight LIVE/DEAD stain [Life Technologies; 0.5 μl/ml Syto-9 and 1.5 μl/mL propidium iodide (PI) in 0.85% (wt/vol) NaCl] was applied to each well and allowed to incubate for 30 minutes at room temperature. Stain was then removed and replaced with 200 μl 0.85% NaCl. CLSM was performed on an inverted Zeiss LSM Pascal equipped with an argon laser (488 nm excitation, 505–530 nm bandpass emission for Syto-9, 560 nm longpass emission for PI) at 400× magnification. Confocal z-stacks were acquired at 1 μm intervals from random fields of view located in the middle of each well. Biofilm parameters (biomass, roughness co-efficient) were quantified using COMSTAT [34].

### CLSM of pre-formed *S*. *mutans* biofilms treated with P85 micelles

Overnight cultures of *S*. *mutans* UA159 were diluted to an OD_600_ of 0.01 in BM containing 0.25% glucose, 0.25% sucrose and transferred to an optically-clear tissue-culture plate as described above. After 24-hours of static growth at 37°C, 5% CO_2_, media was carefully removed from each well and then fresh BM media containing 5 μl of P85, P85F, or F solution (described above) was added to each well. BM containing P85, P85F, or F was again replaced at 48 and 72 hours growth. At 96 hours growth, biofilms were stained with LIVE/DEAD stain and imaged by CLSM as described above.

### Quantification of biofilm viability

To quantify biofilm viability, a parallel series of experiments was performed as described above (treatment of sucrose-dependent or sucrose-independent biofilms at the time of inoculation, and treatment of preformed biofilms). For time-of-inoculation biofilms, the culture supernatant of each well was removed at 48 hours growth, and the adherent biofilms were scraped and resuspended in 200 μl sterile THYE, and then transferred into a sterile microcentrifuge tube. Each tube was then vortexed at high-speed for 15 seconds, followed by 1:10 serial dilution in sterile THYE, and track plating on square THYE plates [35]. After 48 hours growth in 5% CO_2_ and 37°C, the corresponding CFU/biofilm was calculated. CFU determination of preformed biofilm experiments was conducted as described above, except that the biofilms were harvested at 96 hours growth.

### Statistical analysis

For all COMSTAT measurements and CFU/ml determinations, Sigmaplot software (version 12.5, build 12.5.0.38) was used to perform statistical analysis. Data was subjected to normality and equal variance tests, followed a one-way ANOVA and appropriate parametric or non-parametric pairwise test to detect significant differences between treatment groups.

## Results

### *S*. *mutans* biofilms display altered structure and increased cell death when cultured in the presence of farnesol-containing NBMs

**Fig 1:**
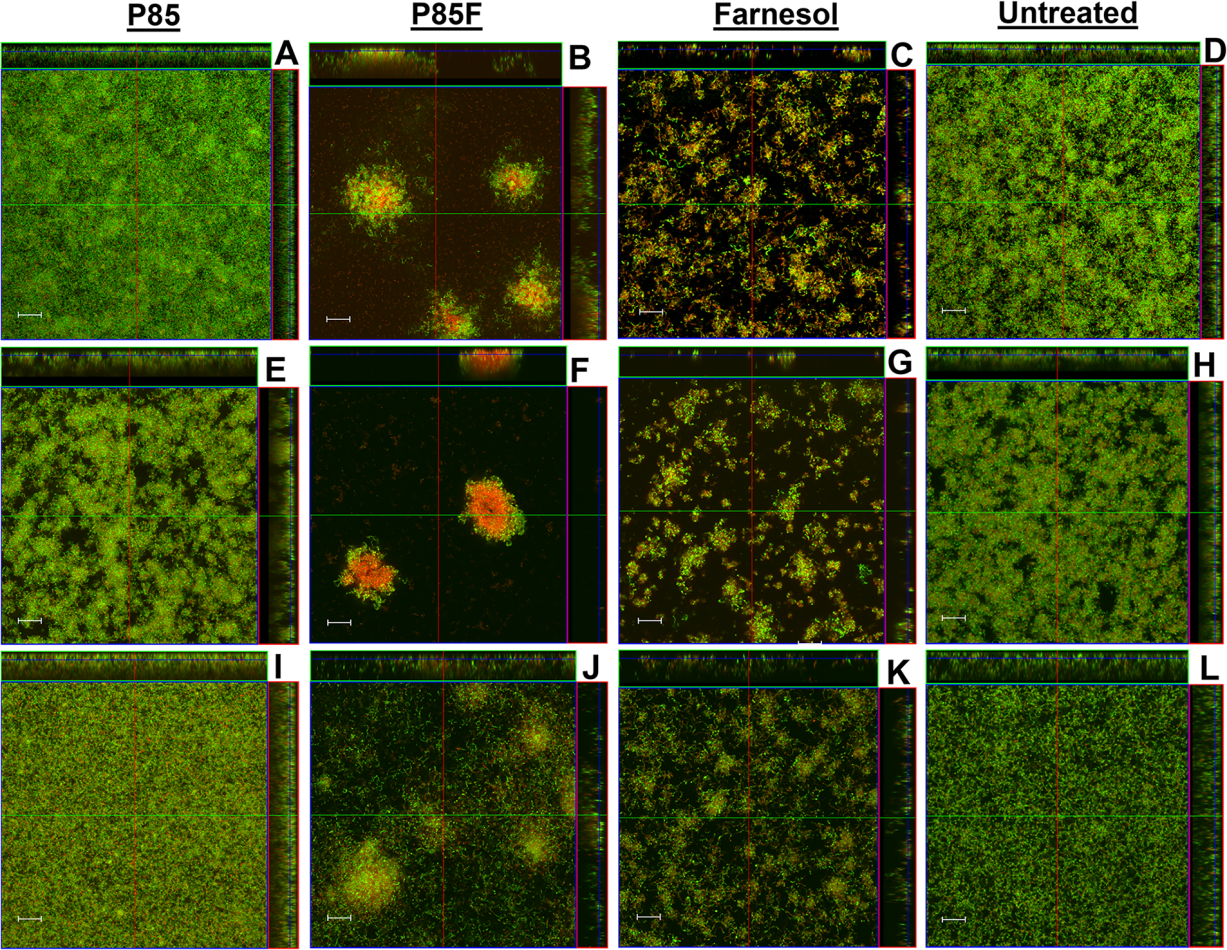
Morphology of *S*. *mutans* biofilms grown with Pluronics-formulated farnesol. Strains UA159 (A-D), BM71 (E-H), and P1 (I-L) static biofilms were grown in BM containing 0.25% glucose and 0.25% sucrose, either untreated or in the presence of 2% P85 (P85), 2% P85 formulated with 50 μg/ml farnesol (P85F), or 50 μg/ml farnesol alone (F). After 48 hours of growth at 37°C in 5% CO_2_, wells containing adherent biofilm were stained with LIVE/DEAD stain (Life Technologies), whereby live cells fluoresce green and dead/damaged cells fluoresce red. Biofilm z-stacks were acquired at 400× magnification by CLSM. Orthogonal images of each biofilm are shown, representing 12 random fields of view from 3 biological replicates acquired over 2 independent experiments. Scale bars = 20 μm.

**Fig 2:**
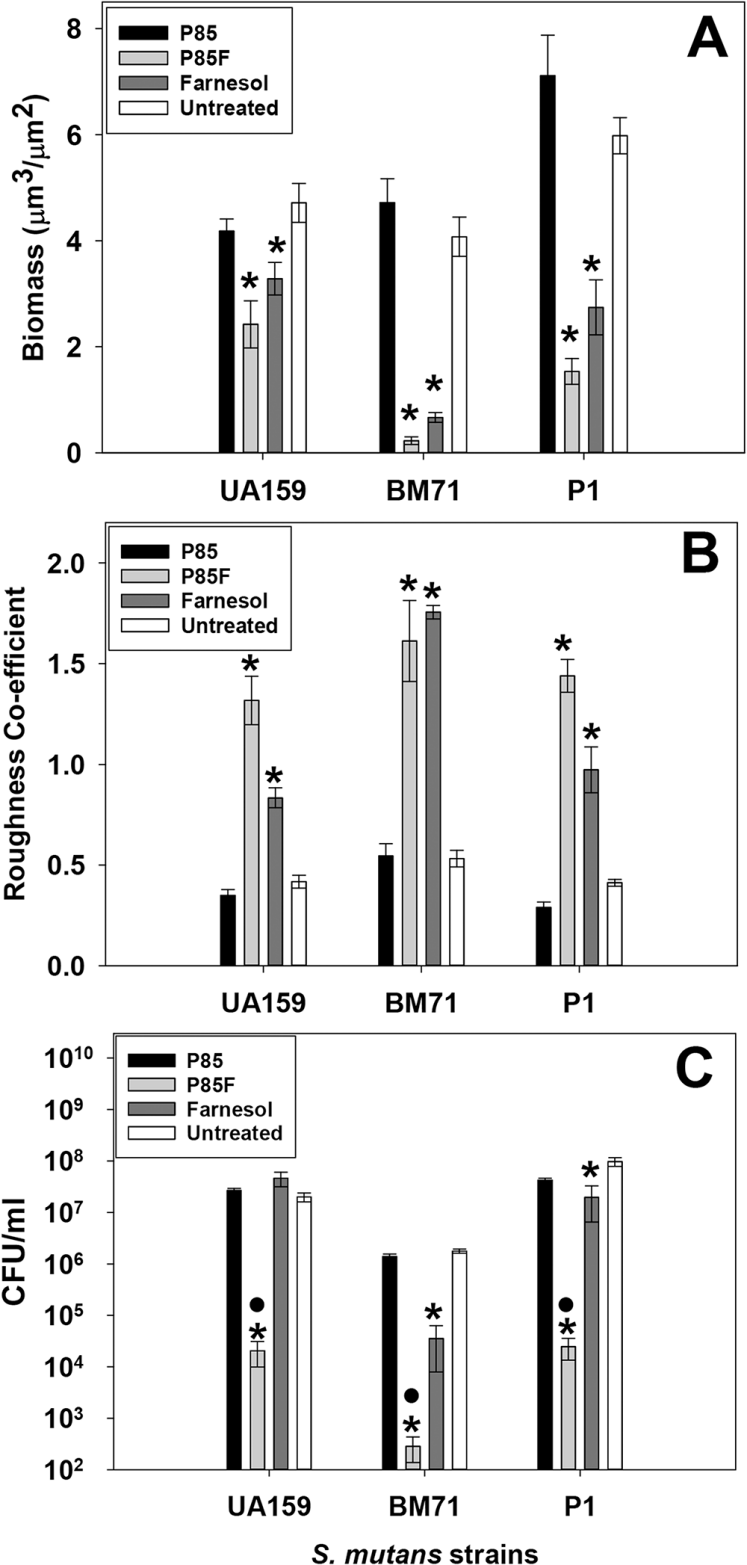
Quantification of P85-formulated farnesol effects on *S*. *mutans* biofilm architecture and viability. Biofilms described in Fig 1 were analyzed with COMSTAT software [34] to quantify CLSM z-stack images for biomass (A) and roughness co-efficient (B). Viability (CFU/ml) of disrupted biofilms was determined by serial dilution plating (C). For A and B, data = 12 random fields of view from 3 biological replicates acquired over 2 independent experiments. The average CFU/ml of biofilms grown in the presence of each condition (Panel C) was averaged from 3 biological replicates acquired over 2 independent experiments. Error bars = standard error of the mean (SEM). *Denotes statistically-significant difference relative to untreated condition (p < 0.05); •Denotes statistically-significant difference compared to farnesol-treated condition (p < 0.05). A: Holm-Sidak test (UA159) or Dunnett’s test (BM71 and P1); B: Dunnett's test; C: Student-Newman-Keuls (SNK) test.

**Fig 3:**
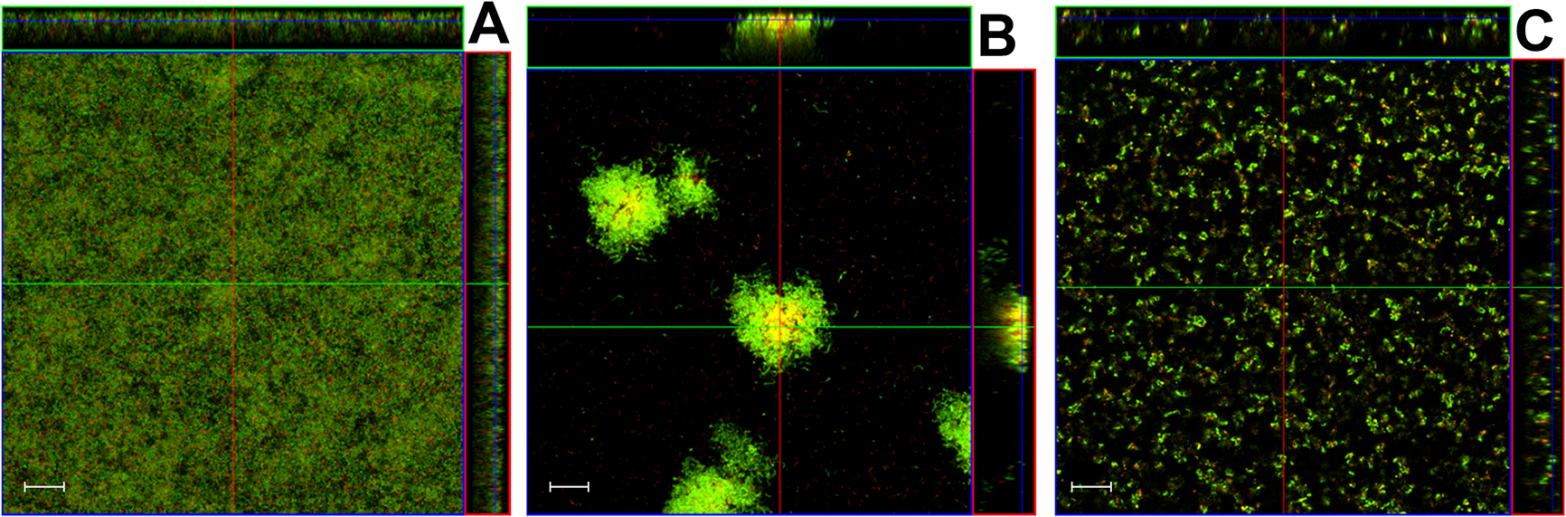
Farnesol-specific tower formation in *S*. *mutans* static biofilm. *S*. *mutans* UA159 static biofilms were grown in BM media containing 0.25% sucrose and 0.25% glucose, either with 2% P123 alone (A) or in the presence of 2% P123 formulated with farnesol (B) or triclosan (C). After 48 hours of growth, wells containing adherent biofilm were stained with LIVE/DEAD stain, and biofilm z-stacks were acquired at 400× magnification by CLSM. Representative orthogonal images of each biofilm are shown, representing 8–12 random fields of view. Scale bars = 20 μm.

Although previous studies have demonstrated the effectiveness of antimicrobial-formulated Pluronic micelles in inhibiting growth of *S*. *mutans* biofilms [26–28], their contribution to biofilm architecture and physiology has not been investigated. Therefore, CLSM was employed in this study to observe changes in biofilm morphology associated with growth in the presence of farnesol-containing Pluronic micelles. Altered biofilm structure was observed in all three tested *S*. *mutans* strains when grown in media containing P85F (Fig 1B, 1F and 1J) or F alone (Fig 1C, 1G and 1K), whereas biofilms grown in the presence of P85 alone (Fig 1A, 1E and 1I) were similar to untreated biofilms (Fig 1D, 1H and 1L). Specifically, growth in the presence of P85F resulted in the formation of large towers containing a centralized area of dead or damaged cells surrounded by live viable bacteria, whereas growth with F alone resulted in a less densely-packed biofilm lacking tower formation, and contained a mixture of live and dead cells throughout the biofilm. *S*. *mutans* cells also tended to form longer chains in the F alone-treated biofilms. These qualitative biofilm characteristics were quantified using COMSTAT software, with both P85F and F biofilms of all three *S*. *mutans* strains displaying significant decreases in biomass (Fig 2A) and increases in roughness co-efficient (Fig 2B) compared to untreated and NBM biofilms. To determine if tower formation was dependent on the specific Pluronic (P85) and/or antimicrobial (farnesol) used, CLSM experiments were repeated on UA159 biofilms grown in the presence of P123 formulated with farnesol (P123F) as well as P123 formulated with triclosan (P123T). These results showed that tower formation occurred in biofilms grown with P123F (Fig 3B) but not in biofilms grown with P123T (Fig 3C) or P123 alone (Fig 3A), suggesting that tower formation is specific to farnesol-containing Pluronic micelles.

Cell viability in all biofilms that were untreated or grown with P85, P85F or F alone was also measured in parallel experiments by serial dilution and CFU plating of disrupted biofilms (Fig 2C). This analysis revealed that biofilms grown in the presence of P85F underwent a 3-log reduction in viability in strains UA159 and P1, and a 4-log reduction in viability in strain BM71. By comparison, biofilms grown in F alone underwent a 1.5-log decrease in viability in BM71, 0.5-log decrease in P1, and no change in viability in UA159 (Fig 2C), indicating that the degree of biofilm killing by farnesol alone may be somewhat strain-dependent. These results demonstrate that in addition to being significantly more effective at killing *S*. *mutans* biofilm relative to farnesol alone, Pluronics-formulated farnesol induces dramatic alterations in biofilm architecture.

### NBMs containing farnesol can alter biofilm structure and promote cell death in pre-formed biofilms

**Fig 4:**
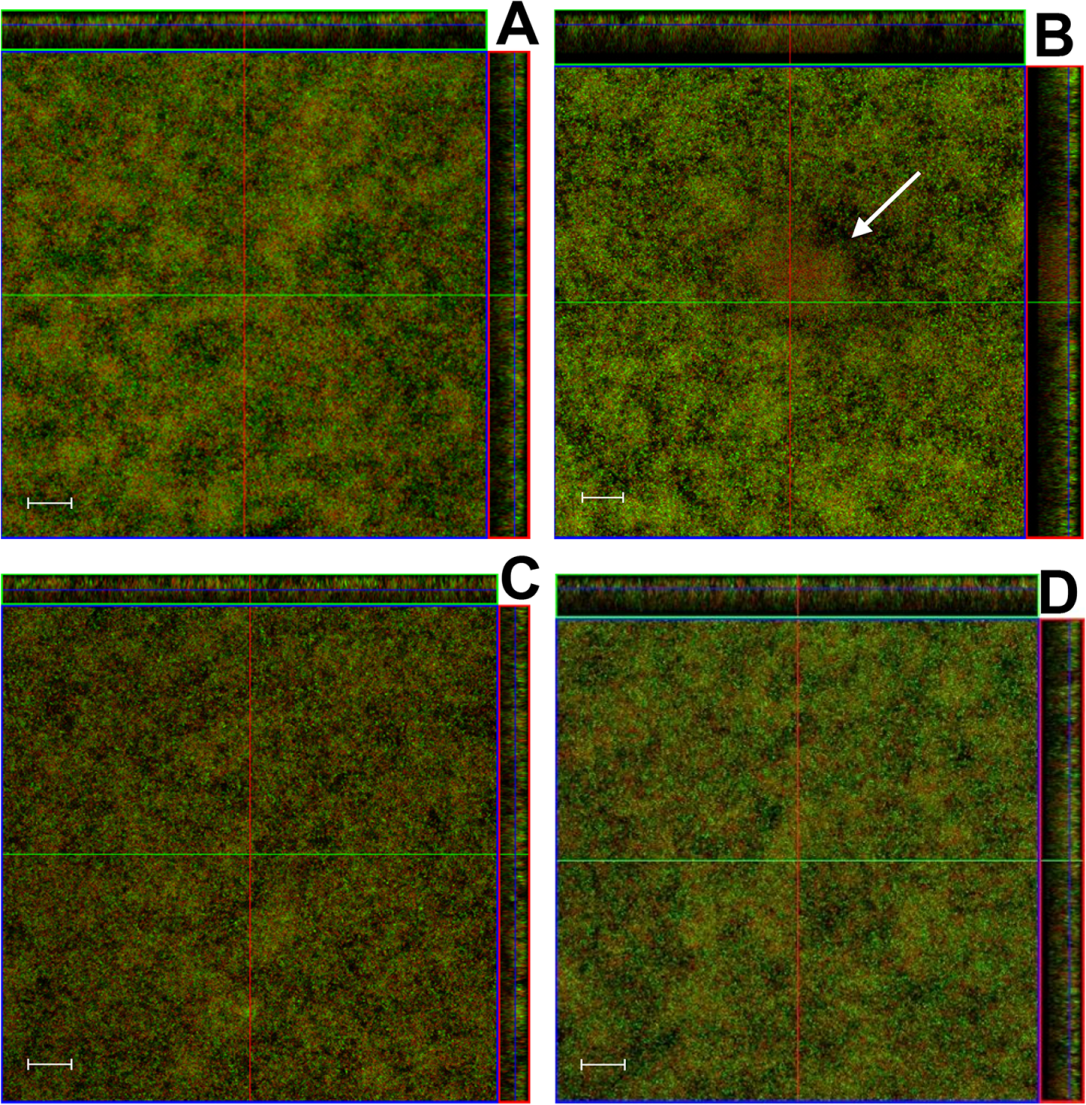
Effect of pluronics-formulated farnesol treatment on pre-formed biofilm architecture. UA159 pre-formed biofilms were treated with P85 (A), P85F (B), F alone (C), or left untreated (D) at 24, 48, and 96 hours growth in 5% CO_2_ at 37°C. After 96 hours, the supernatant was removed and adherent biofilms were stained with LIVE/DEAD stain. Biofilm z-stacks of 1 μm cross-sectional images were acquired at 400× magnification by confocal laser scanning microscopy (CLSM). Orthogonal images of each biofilm are shown, representing 8 fields of view acquired from 2 biological replicates. Scale bars = 20 μm.

**Fig 5:**
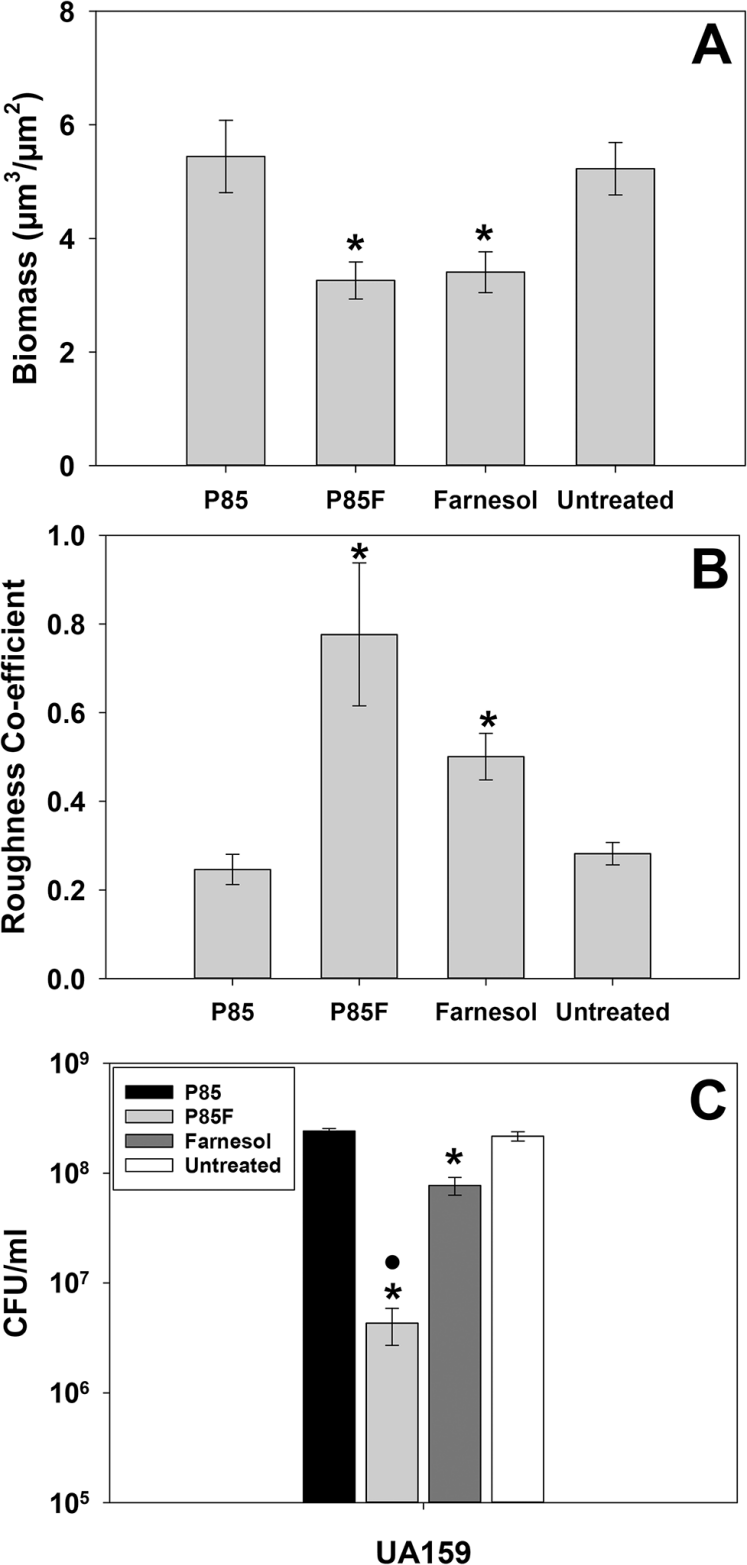
Quantification of P85-formulated farnesol effects on pre-formed *S*. *mutans* biofilm architecture. Biofilms described in Fig 4 (8 random fields of view acquired from 2 biological replicates) were analyzed with COMSTAT software [34] to quantify CLSM z-stack images for biomass (A) and roughness co-efficient (B). The average CFU/ml (n = 3 biological replicates) of biofilms grown in the presence of each condition was also determined in a parallel experiment (C). Error bars = SEM. *Indicates statistical significance compared to untreated control •Denotes statistically-significant difference compared to farnesol-treated condition (p < 0.05; SNK test).

In addition to inhibition of biofilm formation, farnesol-formulated Pluronic micelles were tested for their effect on viability and architecture of pre-formed biofilms (Figs 4 and 5). Repeated treatment of pre-formed UA159 biofilms with either P85F or F alone (Fig 4B and 4C) over a three day period resulted in biofilms with decreased biomass (Fig 5A) and increased roughness (Fig 5B) relative to biofilms treated with P85 alone or untreated biofilms (Fig 4A and 4D). Furthermore, formation of cell death towers was also sporadically observed in pre-formed biofilms treated with P85F (Fig 4B), whereas pre-formed biofilms treated with F alone contained an evenly-distributed mixture of live and dead cells throughout the biofilm (Fig 4C). In parallel experiments, pre-formed biofilms treated with P85F experienced a 2-log decrease in cell viability relative to untreated biofilms, and treatment with F alone only reduced biofilm viability by 0.5-log (Fig 5C). These results demonstrate that tower formation and significant cell death are features common to *S*. *mutans* biofilms treated with P85F.

### Tower formation is dependent on the presence of sucrose-dependent biofilm matrix

**Fig 6:**
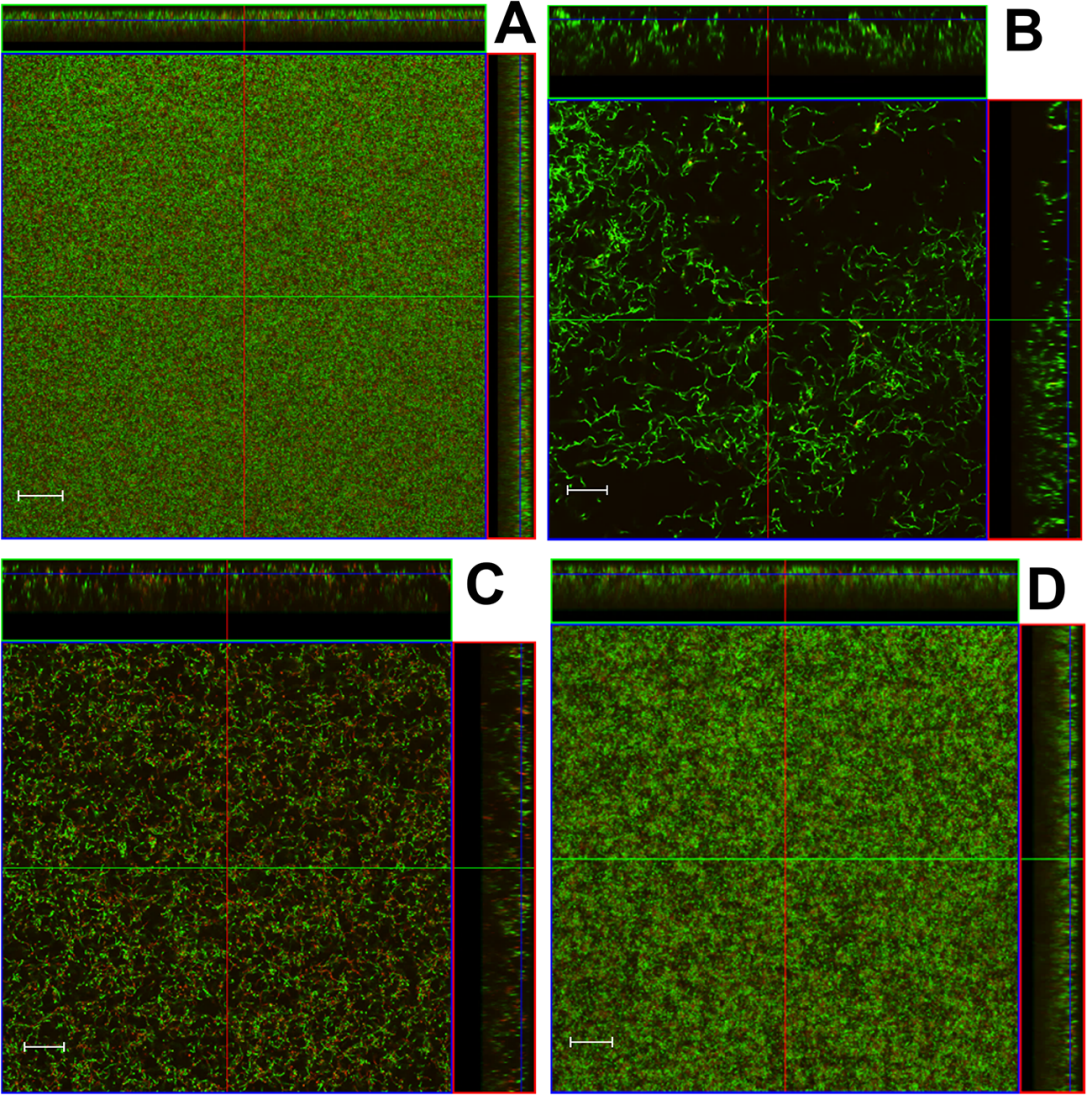
Effect of P85-formulated farnesol on *S*. *mutans* sucrose-independent biofilm architecture. UA159 static biofilms were grown in BM lacking sucrose (supplemented with 0.5% glucose) in the presence of P85 (A), P85F (B), F alone (C), or untreated (D). After 48 hours of growth, adherent biofilm was stained with LIVE/DEAD stain. Biofilm z-stacks were acquired at 400× magnification by CLSM, and orthogonal images are representative of 12 random fields of view acquired from 2 independent experiments. Scale bars = 20 μm.

**Fig 7:**
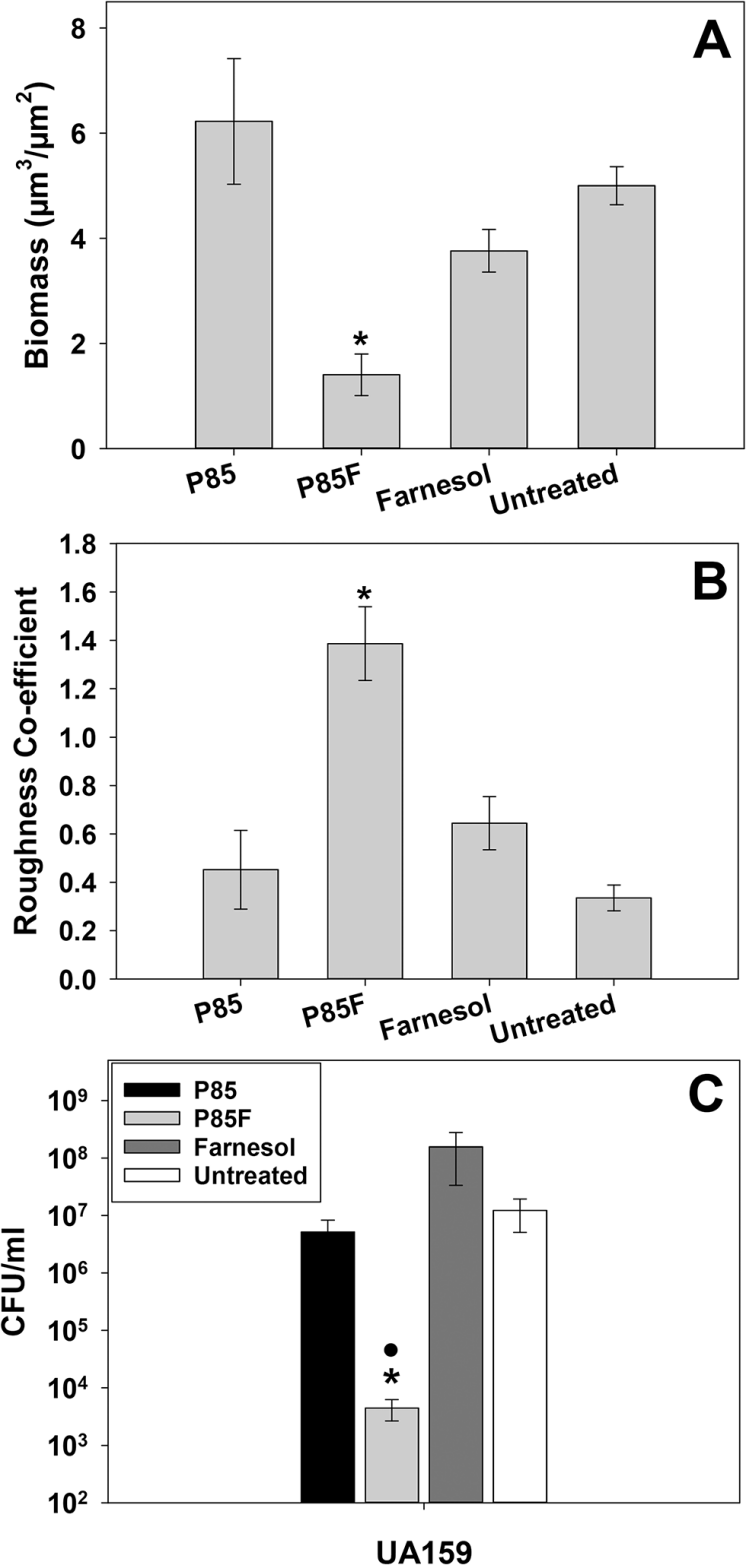
Quantification of P85-formulated farnesol effects on sucrose-independent *S*. *mutans* biofilms. Biofilms described in Fig 6 (12 random fields of view acquired from 2 independent experiments) were analyzed with COMSTAT software [34] to quantify CLSM z-stack images for biomass (A) and roughness co-efficient (B). The average CFU/ml (n = 3 biological replicates, representative of two independent experiments) of biofilms grown in each condition was also determined in a parallel experiment (C). Error bars = SEM. *Indicates statistical significance compared to untreated control •Denotes statistically-significant difference compared to farnesol-treated condition (p < 0.05; SNK test).

*S*. *mutans* produces an EPS biofilm matrix primarily composed of a glucan polymer when grown in the presence of sucrose, and production of this sucrose-dependent matrix is dependent on GTF enzymes [36–38]. To determine if formation of towers and/or increased cell death of P85F treated *S*. *mutans* biofilms was dependent on production of the sucrose-dependent biofilm matrix, UA159 biofilms were grown in media lacking sucrose in the presence of NBM, P85F, and F. Although formation of “death towers” was not observed in these P85F sucrose-independent biofilms (Fig 6B), they displayed decreased biomass (Fig 7A) and increased roughness (Fig 7B) relative to untreated biofilms (Fig 6D), biofilms treated with P85 (Fig 6A) or farnesol alone (Fig 6C). Additionally, *S*. *mutans* cells in both the P85F and F alone biofilms (Fig 6B and 6C) each displayed the characteristic increased cell chain length and patchy biofilm morphology that was observed in F alone biofilms grown in sucrose-containing media (Fig 1C).

**Fig 8:**
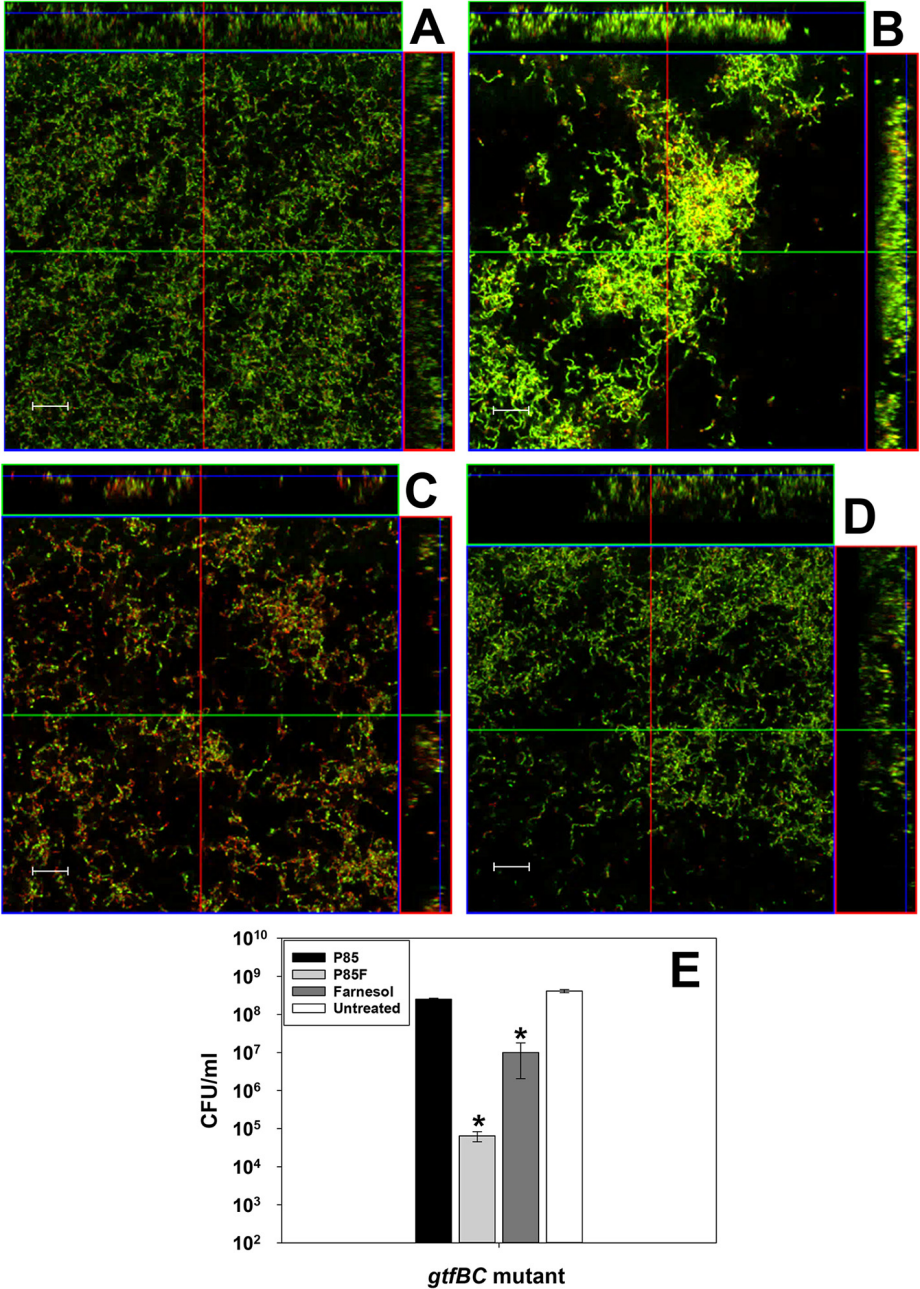
Effect of *gtfBC* mutation on *S*. *mutans* biofilm architecture and viability in response to P85-formulated farnesol. *S*. *mutans gtf*BC mutant was grown for 48 hours in the presence of P85 (A), P85F (B), F alone (C) or untreated (D) in BM media containing 0.25% sucrose and 0.25% glucose. Wells containing adherent biofilm were stained with LIVE/DEAD stain. Biofilm z-stacks of 1 μm cross-sectional images were acquired at 400× magnification by CLSM. Representative orthogonal images of each biofilm are shown. Scale bars = 20 μm. In a parallel experiment (n = 3 biological replicates), the average CFU/ml of each biofilm was also determined (E). All data are representative of 2 independent experiments. Error bars = SEM. *Denotes statistically-significant difference relative to untreated condition (p < 0.05), SNK Test.

To independently verify that P85F-induced tower formation does not occur in the absence of sucrose-dependent biofilm matrix, this experiment was repeated on *S*. *mutans gtfBC* mutant biofilms grown in the presence of sucrose (Fig 8). As predicted, tower formation was not observed in *gtfBC* mutant biofilms grown in the presence of sucrose and P85F (Fig 8B), confirming a role for the glucan biofilm matrix in promoting P85F induced tower formation. Growth of either sucrose-independent UA159 biofilms (Fig 6A) or sucrose-dependent *gtfBC* mutant biofilms (Fig 8A) in the presence of P85 alone did not alter their biofilm structure relative to corresponding untreated biofilms (Figs 6D and 8D).

Surprisingly, P85F was still able to induce a 3-log reduction in UA159 biofilm viability in media lacking sucrose, whereas treatment with F or P85 alone had no effect (Fig 7C). These results are nearly-identical to the viability patterns observed for UA159 biofilms grown in media containing sucrose (Fig 2C). Similar patterns of viability were also observed in *S*. *mutans gtfBC* mutant biofilms grown in media containing sucrose (Fig 8E), where P85F and F alone biofilms experienced 3-log and 1-log reductions in viability, respectively, compared to untreated biofilms. Collectively, these results suggest that although tower formation is dependent on *S*. *mutans* production of a sucrose-dependent biofilm matrix, the formation of towers is not required for increased killing of *S*. *mutans* biofilms by P85F.

## Discussion

Pluronic copolymers are FDA-approved and have been used to efficiently formulate various drugs for their delivery to the stomach [39], brain [40, 41], liver [42], and various tumor cells [43–47]. Additionally, Pluronics conjugated to alendronate, pyrophosphate, and phosphoserine have shown great promise as tooth-targeting systems when used in the micellar formulation of hydrophobic antimicrobial compounds [26–28]. These seminal *in vitro* studies demonstrated that tooth-binding Pluronics micelles loaded with either triclosan or farnesol were much more effective at inhibiting *S*. *mutans* biofilm growth as well as killing pre-formed *S*. *mutans* biofilms grown on HA discs, compared to treatment with each respective free antimicrobial. More recently, this drug delivery principle has been elaborated by others to the use of nanoparticles consisting of a cationic corona and a hydrophobic and pH-responsive core, which exhibited high-affinity targeted binding to the tooth surface, pellicle, and biofilm EPS [48]. When applied topically to the tooth surface twice-daily, these farnesol-loaded nanoparticles were shown to significantly reduce the number and severity of carious lesions in a rat model of *S*. *mutans*-initiated dental caries, relative to treatment with farnesol alone [48]. Up to this point, the anti-caries properties of unformulated farnesol had demonstrated very limited effectiveness against *in vitro* and *in vivo S*. *mutans* biofilms [49, 50], likely due to the poor solubility of farnesol in aqueous solution and limited retention on the tooth surface.

The above-mentioned studies clearly illustrate the powerful translational potential of targeted antimicrobial delivery as an effective treatment strategy against dental caries. However, a challenge to eventual clinical approval of these delivery systems is validation of the safety profiles of newly developed formulation excipients. For example, although alendronate is used clinically in the treatment of osteoporosis [51, 52], there is concern regarding the potential long-term impact of bisphosphonates on bone resorptive activity and its rare association with osteonecrosis of the jaw [53, 54]. Therefore, use of GRAS (Generally Regarded as Safe) excipients that have already been approved for use in humans (including unmodified Pluronic copolymers) as antimicrobial delivery vehicles may represent a relatively streamlined approach for caries treatment. In this respect, the results reported in this manuscript, along with previously-published observations by our group [26–28] suggest that unmodified Pluronics also show great potential as alternative excipients. P85-formulated farnesol was found to both significantly inhibit biofilm growth when added to culture at time of inoculation, as well as efficiently kill established *S*. *mutans* biofilms (Figs 2 and 5). Furthermore, P85-formulated farnesol was able to effectively inhibit biofilm growth of three different *S*. *mutans* strains, suggesting that use of this treatment should be effective against the wide variety of genetically-diverse *S*. *mutans* isolates encountered in the human oral cavity.

Farnesol was chosen as the antimicrobial focus in these studies because (1) it has already been approved for use as a fragrance ingredient in topical cosmetics, shampoos, soaps, and perfumes [55]; (2) it is a naturally-occurring isoprenyl alcohol product found in propolis (a beehive product), fruits and vegetables [56]; (3) it has been shown to exhibit low toxicity in mice and rats [55, 57, 58]; and (4) farnesol has been previously shown to act as a membrane-active antimicrobial with significant inhibitory activity against *S*. *mutans* planktonic cultures [59, 60]. Farnesol has been shown to inhibit growth of multiple bacterial species including *Staphylococcus aureus* [61, 62], *S*. *epidermidis* [63], and *S*. *mutans* [49, 60], by targeting the membrane and increasing hydrogen permeability into the cell [59]. As well, *Candida albicans* synthesizes farnesol as a quorum sensing molecule [64–66] that can trigger apoptosis of this organism when present at high levels [67–69]. Interestingly, our results demonstrate that treatment of *S*. *mutans* biofilms with Pluronics-formulated farnesol results in “death” tower formation. This phenomenon was independent of the type of Pluronic used, but was specific to formulated farnesol (i.e. Pluronics-formulated triclosan did not induce tower formation). This suggests that Pluronics-formulated farnesol interacts with the *S*. *mutans* biofilm in a manner distinct from farnesol alone. Although the specific molecular mechanism behind this interaction was not identified in this current study, our data suggest that the sucrose-dependent biofilm EPS is likely involved (Figs 6and 8).

Farnesol alone also induced alteration of *S*. *mutans* biofilm structure in this study, whereby cells formed longer chains relative to untreated biofilms. Farnesol treatment of other microbial biofilms has been shown to induce changes in biofilm development and/or EPS production. For example, exposure of pre-formed *S*. *epidermidis* biofilms to high levels (300 μM) of farnesol resulted in significantly increased EPS production, reduced biofilm thickness, and altered biofilm structure with increased numbers of dead/damaged cells [70, 71]. A similar result was reported for *S*. *aureus*, where growth in the presence of farnesol had an inhibitory effect on biofilm formation and viability [72, 73]. At high concentrations (300 μM), farnesol has also been shown to prevent germination of adherent *C*. *albicans* cells, whereas at lower farnesol concentrations, these biofilms displayed increased density and were comprised of a mixture of yeast cells, pseudohyphae, and true hyphae [74].

The results reported in this study have also demonstrated that the killing efficacy of Pluronics-formulated farnesol is unrelated to the tower formation that occurs in *S*. *mutans* sucrose-dependent biofilms. Specifically, sucrose-independent wild-type biofilms grown in the presence of P85F did not form towers but still displayed a 3-log reduction in viability compared to the untreated control (Figs 6 and 7). Likewise, *gtfBC* mutant biofilms grown in the presence of sucrose and P85F also did not form towers but displayed a 3-log reduction in viability (Fig 8). Dissection of the cellular changes that precede tower formation and cell death in these biofilms will be the subject of future study in our lab, in addition to determining if tower formation affects the interaction of *S*. *mutans* with other bacterial members of dental plaque. This information will be critical in pursuing the use of unmodified Pluronics formulated with farnesol as a viable strategy in the treatment and prevention of dental caries.
